# How neurotypical listeners recognize emotions expressed through vocal cues by speakers with high-functioning autism

**DOI:** 10.1371/journal.pone.0293233

**Published:** 2023-10-24

**Authors:** Mindy T. Gibson, Maren Schmidt-Kassow, Silke Paulmann

**Affiliations:** 1 Department of Psychology and Centre for Brain Science, University of Essex, Colchester, United Kingdom; 2 Department of Psychiatry, University Hospital, Goethe University Frankfurt, Frankfurt, Germany; Università degli Studi di Bari: Universita degli Studi di Bari Aldo Moro, ITALY

## Abstract

We conducted an investigation to explore how neurotypical (NT) listeners perceive the emotional tone of voice in sentences spoken by individuals with high-functioning autism spectrum disorders (ASD) and NT speakers. The investigation included both male and female speakers from both groups. In Study 1, NT listeners were asked to identify the emotional prosody (anger, fear, happiness, surprise or neutral) conveyed by the speakers. Results revealed that emotional expressions produced by male ASD speakers were generally less accurately recognized compared to male NT speakers. In contrast, emotions expressed by female ASD speakers were more accurately categorized compared to female NT speakers, except when expressing fear. This suggests that female ASD speakers may not express emotional prosody in the same way as their male counterparts. In Study 2, a subset of produced materials was rated for valence, voice modulation, and voice control to supplement Study 1 results: Female ASD speakers sounded less negative when expressing fear compared to female NT speakers. Male ASD speakers were perceived as less positive than NT speakers when expressing happiness. Voice modulation also differed between groups, showing a tendency for ASD speakers to follow different display rules for both positive emotions (happiness and surprise) tested. Finally, male ASD speakers were rated to use voice cues less appropriately compared to NT male speakers, an effect less pronounced for female ASD speakers. Together, the results imply that difficulties in social interactions among individuals with high-functioning ASD could be due to non-prototypical voice use of male ASD speakers and emphasize that female individuals do not show the same effects.

## Introduction

Mastering emotional non-verbal behavior is key to successful interactions. For most people, detecting and expressing emotions through speech comes naturally, as indicated by high recognition rates for emotional speech (e.g., [1]). However, autistic individuals show differing patterns in their emotional expression and perception (e.g., [2–6]). The current investigation aims to contribute to the growing literature on how emotions expressed by autistic individuals are perceived by neurotypical (NT) listeners. Specifically, we investigate whether NT listeners have difficulties recognizing emotions expressed by autistic speakers compared to emotions expressed by NT speakers. We also aim to determine if NT listeners can equally recognize emotions expressed by female autistic speakers and autistic male speakers.

### Emotion recognition and expression in ASD

Numerous studies have shown that individuals with autism spectrum disorder (ASD) struggle with interpreting emotions conveyed through vocal cues (e.g., [3, 5, 6]). Additionally, evidence (e.g., [2, 4]) suggests difficulties in expressing social communicative intentions, making it challenging for NT listeners to infer the emotional intention. Autistic speakers often do not conform to the same conventions as NT speakers when using vocal cues such as pitch, loudness, and speech rate. For example, Grossman and colleagues [2] investigated how children and adolescents diagnosed with high-functioning autism (HFA) retold emotionally laden stories compared to an NT group. The results showed that two NT coders rated HFA participants as more vocally expressive than NTs, but also as sounding more awkward. Despite these differences, HFA output was more accurately identified than NT speakers’ output. Similarly, [4] found an increase in pitch range for autistic individuals when repeating emotional prosody. Furthermore, research by [7] demonstrated that autistic children differ from non-autistic children in pitch control abilities, with a faster response to vocal pitch changes associated with poorer social competence as rated by parents. These findings collectively suggest that ASD speakers use prosodic features differently from NT populations, and these differences in prosody production are linked to social difficulties in autism.

### Emotional communication between neurotypical speakers and individuals with autism

These data prompt the question of whether the differences in vocal cue usage have an actual impact on the emotional communication between NT speakers and individuals with autism. We know from studies on NT speakers that emotional prosody adheres to common display rules, such as the tendency for happiness to be expressed with a high pitch compared to neutral speech [8]. When these norms are followed, others can easily recognize the intended emotion of the speaker. Additionally, brain responses indicate that deviations from listeners’ expectations are quickly detected [9]. Event-related brain potentials specifically demonstrate that listeners detect such violations within approximately 450 milliseconds of a change in vocal expression. Therefore, not conforming to a listener’s expectations may result in communication difficulties. However, despite substantial evidence indicating that abnormal prosodic production is characteristic of ASD (see [10] for a comprehensive review), there is still a scarcity of data on how atypical prosodic production affects the perception of NT listeners. One of the few studies that investigated this important issue examined emotional prosody production in autistic *males* and their NT counterparts. Autistic speakers, when compared to NT speakers, spoke at a slower and louder pace. They also exhibited a wider pitch range when expressing emotions but not when using a neutral voice. These examples were judged to sound "less natural" [11]. Similar to the findings of [2], who reported higher emotion recognition for ASD speech when assessed by two NT coders, [4] demonstrated that naive listeners were also better at categorizing vocal examples from autistic speakers compared to NT speakers. These data once again suggest that non-typical use of cues may not directly impact emotion identification itself but rather primarily lead to an increased perception of *oddness* in NT listeners, as overemphasizing cues violates common display rules.

However, not all studies have confirmed that emotional speech produced by individuals with autism is ultimately better recognized. [12] asked 10 listeners to evaluate materials from individuals with HFA and NTs and reported better identification of angry, happy, and neutral speech when produced by NTs, with a trend towards better recognition of sad speech from individuals with autism. These findings imply that successful categorization might depend on the specific target emotion and/or the possibility that overemphasizing certain cues is more advantageous for certain emotions. In contrast to most other studies, [12] also accounted for response biases in judges, which refers to their inclination to preferentially choose one response category over another. They specifically considered the frequency at which each emotional category was selected (e.g., raters attributed happiness and neutral more often than fear or sadness). When examining these results, the only remaining group difference indicated better recognition of sad utterances expressed by individuals with autism.

In summary, reviewing the literature leads to the following conclusions: 1.) Individuals with ASD exhibit distinct qualitative differences in their expression of emotions compared to NT individuals. 2.) These differences do not necessarily lead to inferior recognition of emotions but rather appear to depend on the specific emotion being expressed. 3.) Further research employing more rigorous methodologies (e.g., a larger number of raters and emotional categories, correction for response bias) is necessary to delve deeper into this issue.

### Current study

Our aim was to make a contribution to this objective by combining the strengths of previous studies and addressing gaps in the literature. Firstly, we examined the ability of NT listeners to recognize the emotional intentions of both HFA individuals and NT speakers. We included both *female* and *male* speakers since previous research has predominantly focused on male participants. Although ASD is more commonly diagnosed in males than females [13], testing both groups is crucial due to gender-related differences in autistic characteristics across behavioral and neural domains [14, 15]. Notably, women are often more challenging to diagnose than men [16], which is linked to their stronger masking or camouflaging tendencies and compensatory behaviors. These masking and compensatory efforts help navigate social situations and may involve suppressing and controlling behaviors associated with autism that are deemed inappropriate in a given context or imitating NT behaviors [17]. These types of behaviors are particularly observed in cognitively high-functioning women, especially those whose autism is recognized later in life [18–20]. In the domain of emotional prosody, compensatory strategies might include autistic speakers attempting to closely imitate or replicate "typical" productions that adhere to emotional "display rules" [21]. Autistic speakers may demonstrate an inclination to exaggerate their emotional speech, similar to observations made in actors when they are instructed to portray various emotional voices [22]. This exaggeration facilitates the determination of the intended emotion by the listener.

Secondly, we adopted the approach of [11] by testing a larger number of NT listeners instead of relying on a small group of coders or trained judges. Thirdly, similar to the methodology of [12], we accounted for response biases in our judges. This is crucial in evaluating which emotions might be more challenging for judges to identify accurately. Finally, to gain a comprehensive understanding of how NT listeners perceive emotions expressed by autistic speakers, we delved into voice perception in greater detail, focusing on the perceived valence/affect, voice modulation, and voice control.

## Methods

Participants of both Study 1 (emotional voice recognition) and Study 2 (voice quality perception) gave written informed consent before participation. The project was ethically approved by the University of Essex Science and Health Faculty Ethics Sub-committee (SP1701). All study-related procedures were in accordance with the Declaration of Helsinki.

### Stimulus development

#### Speakers

All speakers were recruited in summer 2017 on a voluntary basis via email and facebook (snowball effect) calling for individuals previously professionally diagnosed with HFA. We aimed to recruit a similar number of speakers reported in past emotional prosody recognition tasks. While studies exploring differences in *listener* groups tend to present one or two speakers who express materials to listeners (e.g., [12, 23]), studies that aim to compare whether listeners respond differently to different *speaker* groups (e.g., age, gender, culture) often present three to four speakers in their studies (e.g., [8, 24]). Our call for speakers resulted in six HFA volunteers (no payment was offered to participants). We asked all speakers to complete an unlabeled copy of the Autism-Spectrum Quotient (AQ; [25]) after the recordings. The instrument was developed to allow for a brief assessment of autism traits. An AQ score of 35 or higher was obtained from all HFA speakers recruited (scores ranging from 32–50 indicate a strong likelihood of HFA or Asperger’s syndrome, see [25]). To match our HFA sample, we then aimed to recruit an additional six speakers with similar background characteristics (e.g., place of origin, age, gender) who had not received a professional diagnosis of HFA. All volunteers filled out an unlabelled version of the AQ and received a score of 21 or less (i.e. within range of a NT = population; [25]). Table 1 lists the characteristics that we aimed to control for. Paired samples t-test confirmed that groups only differed in AQ scores (*p* < .001) but no other variables (all *p*s≥0.15).

**Table 1:** Speaker information.

Speaker Type	Sex	Age	School	Place of Birth/Childhood	AQ	Emoji
ASD	Female	44	18	Cyprus (UK Military Base)	36	5
ASD	Female	43	18	England	39	5
ASD	Female	38	18	England	44	6
ASD	Male	27	16	USA (California)	38	6
ASD	Male	19	14	England	35	6
ASD	Male	18	13	England	38	6
NT	Female	26	18	Guyana (British Colony)	11	6
NT	Female	23	18	England	14	6
NT	Male	30	17	England	11	6
NT	Male	28	16	USA (California)	4	6
NT	Male	23	18	England	21	6
NT	Male	36	18+	England/Wales	16	6

*Note*. Speaker Type refers to ASD or NT individuals; Age information provided in years; School information refers to total years spent in school; Emoji refers to the score received on an emotional recognition task (scored out of six).

#### Procedure

All recordings took place individually. The first author initially explained the recording procedure, followed by a written reiteration just before the recording started. Following instructions, a brief emoji quiz (Iphone emoticons) was administered to ensure each speaker understood which six emotions (anger, fear, happiness, neutral, sad, pleasantl surprise) they were asked to portray. Next, speakers were asked to read through ten semantically neutral sentences (e.g. “The fence was painted brown”) adapted from a previous study [26] to familiarize themselves with the material before the recording began. Emotions were recorded as blocks in the same order and participants were asked to reflect on a time they had experienced the requested emotion, an induction technique used successfully in past studies. No vocal examples were provided. There was no limit to how often a sentence could be spoken. Stimuli were recorded using *Audacity* in a quiet environment. After recordings were completed, speakers were asked to complete an unlabeled copy of the AQ. They were then debriefed. The total run time ranged from 25 to 45 minutes, depending on the length and frequency of breaks.

#### Stimuli selection

From the total 720 raw recordings, samples with extraneous noises (e.g., tapping, laughing, crying), words cut off, misspoken, or mispronounced, were eliminated. After this, we aimed to ensure that all speakers and emotions would be presented equally in the subsequent study. Thus, we removed additional files for those speakers who were left with more than seven samples per emotion based on the running order of sentence recordings to ensure that we included variation in terms of sentence content (crucially, with this selection mechanism, no judgment could be made from authors in terms of sentence quality). Finally, we used Praat [27] to extract standard acoustic cues that allow characterizing the way speakers expressed emotions. Means showed that ASD speakers expressed sentences with a higher mean pitch (9.5 semitones vs. 6.0), spoke more slowly (2.8 seconds per sentence vs. 2.3 seconds), and used a more constrained loudness range (27dB vs. 31.6dB). They also used less energy in high-frequency bands (18.4 dB vs. 23.6 dB), suggesting that NT speakers used more vocal effort when producing materials.

### Study 1: Emotional prosody recognition study

#### Listener participants

A total of 60 volunteer listener participants were recruited. Seven participants were excluded due to self-reported hearing impairments, speaking a native language other than English, mental health/neurological conditions, or because they failed to provide demographics. Specific data on socioeconomic status and educational attainment levels were not recorded. A total of 53 participants (24 female; age range: 18–70 years; *M* = 36.8, *SD* = 18.3) were included in the final within-subjects analysis. The mean AQ score (Baron-Cohen et al., 2001) of listeners was 16.89 (SD = 6.7). All but one participant (AQ = 38) scored lower than 32 on the questionnaire. The authors did not have access to personally-identifying information.

#### Materials

Seven sentences were spoken by each of the twelve speakers for each emotional category, leaving 504 files for the recognition task. To decrease the task length, only a subset of these files were presented: materials were distributed over two lists, with the experiment software pseudo-randomly choosing 210 files (35 per emotion) from the material sample pool within that list. The exemplars for emotional stimuli and the speakers varied but were balanced overall (autistic and non-autistic speakers occured equally often; emotional categories were presented equally often). The software selected exemplars to avoide experimenter bias. For each listener, the experimental version of the lists was randomized. The recognition task was programmed using *Inquisit* (Millisecond Software).

#### Procedure

Listeners were invited to participate in an online emotional prosody recognition task. Instructions requested participants to be in a quiet setting in which they could complete the 30-minute-long study without distractions. Using a forced-choice response method, participants were instructed to use their best judgment to select the speaker’s intended emotion. Each participant had six practice trials. At the end of the task, participants were asked to fill out an unlabeled version of the AQ questionnaire.

### Study 1 data analysis

The statistical analysis was run on SPSS 24 (IBM software). For the analysis, Wagner’s unbiased hit rate was used to calculate accurate responses to statistically control for biases toward selecting a specific ‘default’ emotion for exemplars [28]. It is a metric used to evaluate the accuracy or reliability of results in a study. The higher the hit rate, the more accurate and reliable the identifications are (1 = highest systematic selection; 0 = represents no systematic selection). Unbiased hit rates were arcsine transformed, a procdeure commonly applied to stabilize the variances and improve the normality of data that represents proportions or percentages. Arcsine-transformed unbiased hit rate data were analyzed using a General Linear Model treating *Speaker Type* (ASD or NT), *Speaker Sex* (female, male), and *Emotion* (angry, fearful, happy, neutral, sad, [pleasantly] surprised) as within-subjects variables.

### Study 1 results

**Fig 1:**
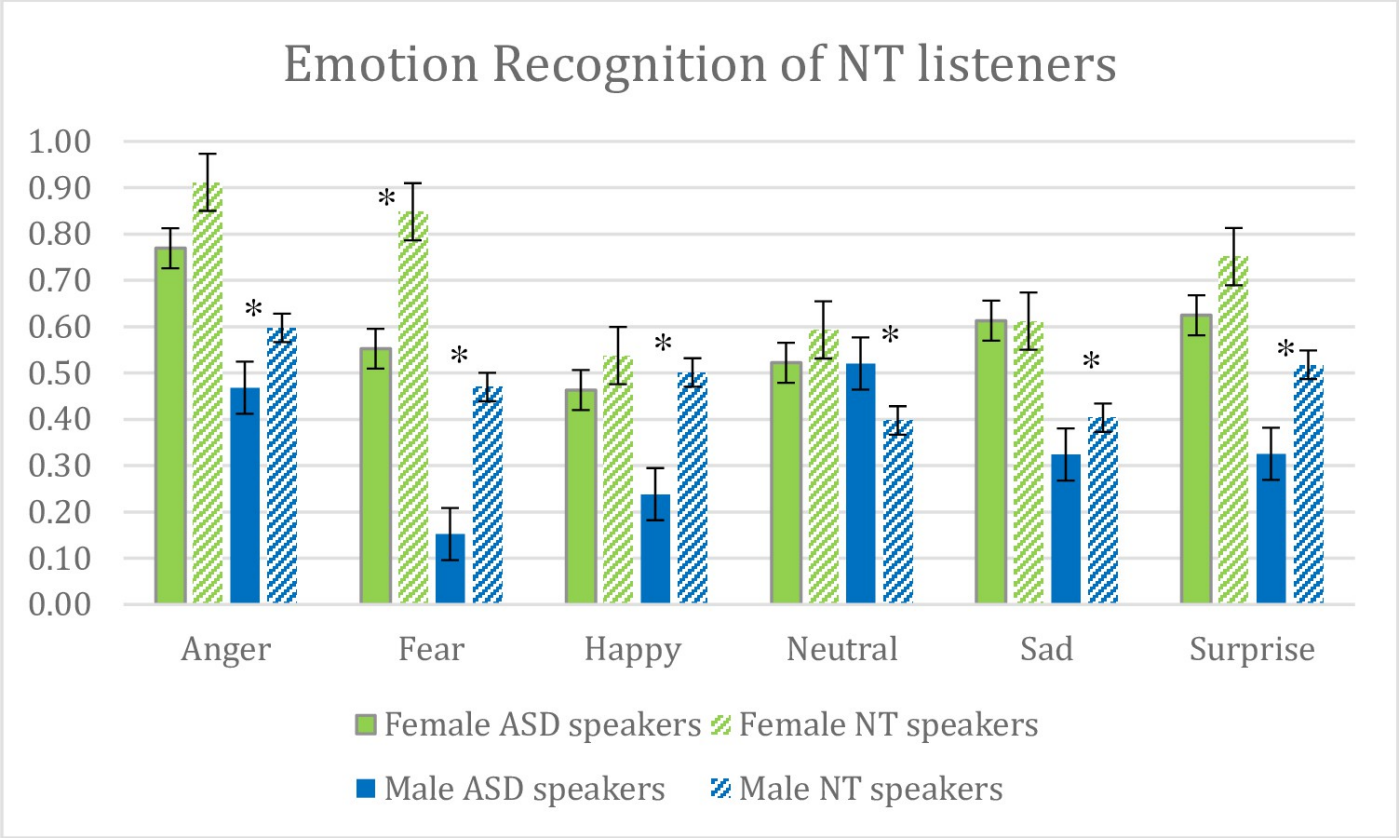
This figure depicts emotion recognition scores (as arcsine transformed Hu scores) obtained from neurotypical (NT) participants when listening to emotional sentences expressed by female and male high-functioning autistic (ASD) and neurotypical (NT) speakers. Error bars reflect standard error. * indicate significant differences at *p* < .05.

Unbiased hit rates were highest for angry vocalizations, followed by pleasantly surprised. Recognition for fearful, happy, neutral and sad exemplars varied depending on speaker type (see Fig 1).

The main effect of *Speaker Sex*, *F*(1, 52) = 262.297, *p <* .001, ηp2 = .835, *Speaker Type*, *F*(1, 52) = 21.468, *p <* .001, ηp2 = .292, and *Emotion*, *F*(5, 260) = 27.592, *p <* .001, ηp2 = .347, were significant. Planned post-hoc *t*-tests for the latter effect showed that all emotional comparisons yielded significant effects (all *p*’s ≤.02) apart from contrasts between fearful and neutral, fearful and sad, and neutral and sad stimuli.

The significant interactions between *Speaker Type* x *Emotion*, *F*(5, 260) = 16.016, *p <* .001, ηp2 = .235, and *Speaker Sex* x *Emotion*, *F*(5, 260) = 8.604, *p <* .001, ηp2 = .142, were informed by a significant three-way interaction, *Speaker Type* x *Speaker Sex* x *Emotion*, *F*(5, 260) = 4.943, ηp2 = .087, *p <* .001. Planned pairwise comparisons by *Speaker Sex* and *Emotion* revealed that male NT speakers were significantly better recognized than male ASD speakers (all *p*’s < .001). For female speakers, there was only a difference between speaker groups when listeners had to recognize fearful stimuli (*p <* .001) which were better recognized if spoken by NT speakers. These data showed differences in how well NT listeners recognized emotions expressed by NT and ASD speakers, highlighting speaker sex effects: male ASD speakers were less well recognized by NT listeners irrespective of which emotions ASDs expressed. In contrast, only fearful expressions were less well recognized when spoken by ASD female speakers when compared to recognition rates for materials spoken by female NT speakers.

### Study 2: Valence, voice modulation, and control over voice cue use

In a second study, we explored how the exemplars from both speaker groups are perceived in terms of affection expressed, pitch modulation, and control over the use of voice cues. This is important to gather information on how listeners perceive the speakers’ speech, rather than just focusing on the ability to recognize emotions expressed.

#### Listener participants

Twenty-four participants (13 male) were recruited through a course-credit list to participate in Study 2. One participant did not complete the demographics questionnaire; the mean age of the remaining participants was 19.8 years (range: 18–22 years); all replied “no” to the question if they had been diagnosed with autism. They also self-reported no hearing problems. Specific data on ethnicity, socioeconomic status, and educational attainment levels were not recorded.

#### Materials

We presented a random subset of voice samples from Study 1. Out of 142 files in total, 68 were spoken by ASD speakers. The stimuli were again presented via *Inquisit Web*.

#### Procedure

Answers were provided on a 7-point Likert scale. We highlighted that there were no “right” or “wrong” answers. To assess valence, listeners could indicate how negative (-3) or positive (+3) a voice sounded. For voice modulation, they could say whether the voice sounded very monotone/flat (-3) or very modulated (+3). Finally, for “How appropriately did the speaker control their loudness, speech rate, and pitch?” the answer scale ranged from “voice did not sound very well controlled” (-3) to “voice was used very appropriately” (+3).

### Study 2 data analysis

**Fig 2:**
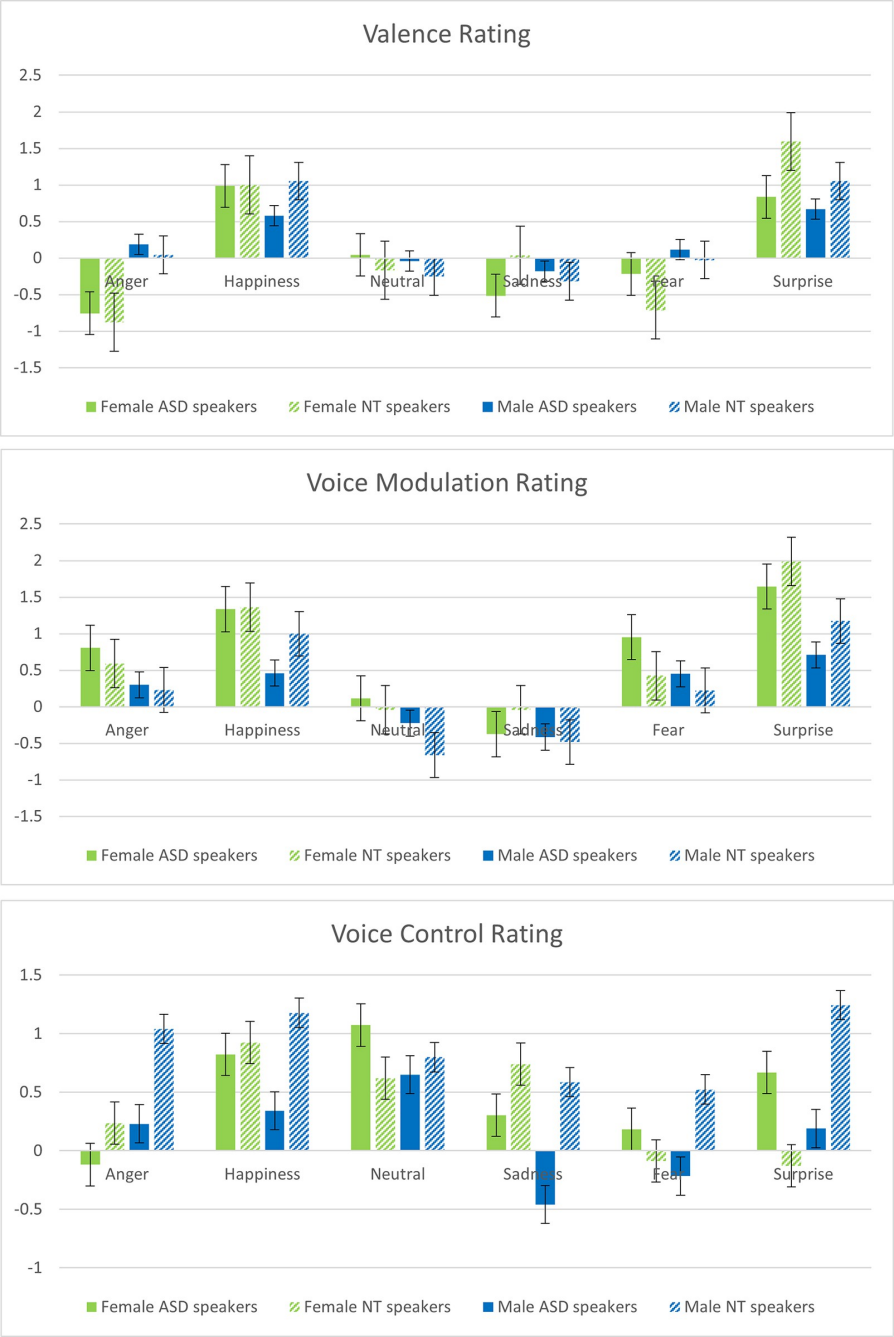
a-c. These figures depict rating scores obtained from neurotypical (NT) participants when listening to a subset of emotional sentences expressed by female and male high-functioning autistic (ASD) and neurotypical speakers. Error bars reflect standard error.

Ratings were aggregated across all stimuli by participants for each dependent variable (DV). A Shapiro-Wilk test was performed for each DV (separately for materials spoken by ASD and NT speakers) and showed that the distribution of scores did not depart significantly from normality for any of the DVs (valence, voice modulation, voice control; all *p*s >.15). Based on this, means of ratings were analyzed with SPSS 24 (IBM software) using a General Linear Model treating *Speaker Type* (ASD or NT), *Speaker Sex* (female, male), and *Emotion* (angry, fearful, happy, neutral, sad, [pleasantly] surprised) as within-subjects variables. Mean ratings for each indicator separately can be found in Fig 2A–2C.

### Study 2 results

#### Valence

Results revealed a significant effect of *Speaker Sex*, *F*(1, 23) = 4. 857, *p <* .05, ηp2 = .174, while *Speaker Type* was not significant (*p* = .27). There was a main effect of *Emotion*, *F*(5, 115) = 42. 678, *p <* .001, ηp2 = .650. Planned post-hoc *t*-tests were conducted to determine which emotions differed from neutral voices, revealing that both positive emotions were rated as sounding more positive than neutral exemplars irrespective of speaker groups. The interactions between *Speaker Sex* x *Emotion* and *Speaker Type* x *Emotion* were both significant (F’s > 9.9 and *p*’s < .001; both ηp2 >.30) as was the three-way interaction between *Speaker Sex* x *Speaker Type* x *Emotion*, *F*(1, 23) = 2. 66, *p <* .05, ηp2 = .104. We followed up the three-way interaction by *Speaker Sex* and *Emotion*. This revealed that female ASD speakers were rated to sound more negative than female NT speakers when expressing sadness (*p* = .001). NT female speakers sounded more negative than ASD female speakers when expressing fear (*p* = .028), and more positive when expressing surprise (*p* = .002). For male speakers differences emerged when looking at ratings for happy expression (*p* = .02), showing NT speakers were rated as sounding more positive than ASD speakers.

#### Voice modulation

A significant main effect of *Speaker Sex*, *F*(1, 23) = 33. 205, *p <* .001, ηp2 = .591, but no main effect of *Speaker Type* (*p* = .949), emerged. There was an effect of *Emotion*, *F*(5, 115) = 33. 183, *p <* .001, ηp2 = .591. Planned post-hoc *t*-tests between neutral and all emotions were significant (all *p*’s < .001) except for sadness, showing that emotions were perceived as sounding more modulated than neutral exemplars irrespective of speaker group. There was a significant interaction between *Speaker Type* x *Emotion*, *F*(5, 115) = 7. 824, *p <* .001, ηp2 = .254. Planned pairwise comparisons indicated differences when expressing fear (*p* = .005) where ASD speakers were rated to modulate their voice more strongly than NT speakers, and for happiness (*p* = .008) and surprise stimuli (*p* < .001) where ASD speakers were rated to modulate their voice less than NT speakers. No such modulation differences were observed for angry, neutral, and sad stimuli. Finally, there was an interaction between *Speaker Sex* x *Emotion*, *F*(5, 115) = 3.565, *p <* .001, ηp2 = .134. Planned posthoc tests revealed that voice modulations from female speakers were perceived to be more pronounced than modulations made by male speakers when expressing anger (*p* = .014), happiness (*p* < .001), neutral (*p* < .001), fear (*p* = .016) and surprise (*p* < .001) but not when expressing sadness. The interactions between *Speaker Sex* x *Speaker Type* and the three-way interaction between *Speaker Sex* x *Speaker Type* x *Emotion* were not significant (*p*’s >.11).

#### Voice control

A significant main effect of *Speaker Type*, *F*(1, 23) = 29.785, *p <* .001, ηp2 = .564, emerged. The main effect of *Speaker Sex* was not significant (*p* = .35) but the main effect of *Emotion* was, *F*(5, 115) = 12. 647, *p <* .001, ηp2 = .355. The interactions *Speaker Sex* x *Speaker Type*, *F*(1, 23) = 29.188, *p <* .001, ηp2 = .559, *Speaker Type* x *Emotion*, *F*(5, 115) = 6.899, *p <* .001, ηp2 = .231, and *Speaker Sex* x *Emotion*, *F*(5, 115 = 6.664, *p <* .001, ηp2 = .225, were informed by a significant three-way interaction between *Speaker Sex* x *Speaker Type* x *Emotion*, *F*(5, 115 = 3.820, *p <* .01, ηp2 = .142, suggesting that listeners perceived differences in terms of voice control between the speaker groups for the different emotions. Planned posthoc tests indicated that for female ASD speakers voice use was perceived to be more “appropriate” than that of female NT speakers when expressing neutral (*p* = .02) and surprise (*p* = .005) but less when expressing sadness (*p* = .02). For male speakers, a different picture emerged. Here, contrasts between ASD and NT speakers were significant for all emotional categories (*p*<001) except for neutral (*p* = .267). In all instances, ASD speakers were perceived to be in less control of their voice.

In short, we report differences between speaker groups in terms of valence, voice modulation, and perceived voice control. Female ASD speakers were perceived as sounding more negative when expressing sadness and less negative when expressing fear when compared to female NT speakers. They were perceived to sound less positive than NT speakers when expressing surprise. Male ASD speakers were perceived as less positive than NT speakers when expressing happiness. ASD speakers were also perceived to be modulating their voice differently from NT speakers when expressing neutral (closer to no modulation), fear (more modulation), happiness, and surprise (less modulation) showing a tendency for different display rules for both positive emotions. Finally, results showed that male ASD speakers were rated to be less in control over speech cues compared to NT male speakers, an effect less pronounced for female ASD speakers.

## General discussion

The primary objective of this investigation was to examine the ability of NT listeners to recognize emotional prosody when produced by individuals with HFA in comparison to their NT counterparts. Investigating potential differences between these groups is crucial as ASD speakers are known to exhibit distinct usage of prosody features [29–31], which may result in perception challenges for NT listeners. Our findings demonstrate that emotional expressions conveyed by NT speakers are generally more accurately recognized by NT listeners than those expressed by individuals with autism. However, in contrast to previous studies, we included an equal number of female and male ASD speakers to capture the full range of emotional vocal displays. Indeed, results showed that female ASD speakers were recognized equally well in comparison to NT female speakers across most emotional categories, except for fear where recognition varied. This indicates that both groups of female speakers utilized acoustic cues consistent with the expectations of emotional exemplars for NT listeners. In contrast, exemplars produced by autistic male speakers were less effectively recognized compared to male NT speakers, except when expressing neutral, where the pattern was reversed. The implications of these results for the existing literature will be discussed below.

### Atypical emotional prosody in male ASD speakers?

It has long been documented that individuals with ASD exhibit distinct prosodic characteristics in their speech compared to NT individuals. Specifically, studies have reported differences in pitch usage, such as higher mean pitch and increased pitch variation, as well as difficulties in controlling loudness (e.g., speaking too loudly or too quietly) in ASD speech [4, 30, 32]. Additionally, ASD speakers tend to have longer utterance durations compared to NT speakers [33, 34]. However, previous studies have primarily focused on acoustic data from male speakers or included a small sample of female speakers within a larger male speaker group (e.g., [30]). Consistent with this body of evidence, the emotional prosody exemplars produced by male speakers here displayed a similar pattern (see S1–S12 Tables). Descriptive analyses suggest that male autistic speakers exhibited higher mean pitch and longer utterance durations compared to male NT speakers. In the case of female speakers, the autistic group showed less variability in loudness and decreased vocal effort (energy in high-frequency regions) when compared to NT speakers. While these descriptive acoustic findings should be interpreted with caution due to the small number of speakers in our study, they provide valuable insights into understanding why emotional prosody produced by female speakers was similarly well recognized across both speaker groups, while male ASD emotional speech exemplars presented perception difficulties (e.g., pitch variations causing confusion for NT listeners rather than reduced loudness variability).

Our results differ from those of [11], who examined male speakers and reported higher recognition rates for stimuli produced by individuals with ASD compared to NT speakers. They suggested that NT listeners found it easier to categorize vocal expressions as ASD speakers exaggerated certain vocal features. Here, we find no such effect, indicating that the acoustic variations observed between our male speaker groups, previously linked to greater expressiveness, did not lead to a more accurate identification of emotional attributes. Instead, the data suggest that deviating from the conventions of NT emotional vocal cues, such as speaking more slowly and with an increased mean pitch, made it more challenging for NT listeners to discern the intended emotion. The observation that happy and surprised expressions sounded more monotone when spoken by ASD speakers compared to NT speakers further supports the notion that emotions were not conveyed in line with NT expectations. Additionally, male ASD speakers were perceived as having less control over their voices. Interestingly, neutral expressions produced by autistic speakers were not less accurately recognized, suggesting that adhering to stereotypical norms may be less crucial in conveying neutrality.

In summary, our findings support the idea that male autistic speakers express emotions differently from their NT counterparts, leading to difficulties for NT listeners in accurately perceiving the intended emotional expression. Importantly, these patterns were not observed for female ASD speakers.

### Typical emotional prosody in female ASD speakers?

Given the past prominent focus on male ASD speakers, it is important to examine the speech of female ASD speakers more closely. Evidence suggests that even girls who do not meet the full criteria for an ASD diagnosis may still exhibit difficulties in social communication contexts [35], suggesting that atypical communication is not solely a characteristic of male autistic speakers. Nevertheless, it remains to be determined how prosody usage differs between male and female ASD speakers and how it compares to NT speakers.

Descriptive results from our study (see S1 Table) indicate that female ASD speakers only differed from female NT speakers in their use of loudness and vocal effort. Thus, the two groups exhibit comparable use of acoustic characteristics for other key variables, resulting in similar recognition rates among NT listeners for sentences expressed by both groups. In contrast to male ASD speakers, female individuals with autism had their emotional intentions accurately recognized by NT listeners in all tested categories, except for the category of fear. This finding is further illuminated by findings from Study 2. Of particular interest is that NT female speakers were perceived as sounding more negative than female ASD speakers when expressing fear, potentially explaining why NT fear expressions were easier to recognize. Furthermore, in terms of voice modulation, listeners rated fearful stimuli expressed by autistic speakers (regardless of gender) as sounding more modulated. This raises the question of whether autistic speakers were attempting to overcompensate in expressing this specific emotion, an effect not observed for other emotions where NT speakers were rated as sounding more modulated.

Building on the idea of over-expressed output [11], we speculate that the excessive use of specific features can impede the recognition of emotions when the produced pattern deviates too much from prototypical displays or creates too much overlap with other emotional intentions, leading to ambiguity between categories. Thus, not fully adhering to prototypical or normative standards (e.g., using a wider pitch range or a louder voice) may hinder the recognition of their expressions at times. However, the effect of "misuse" or "exaggeration" of cues could depend on the relative "importance" of an acoustic cue in expressing the intended emotion. For example, if speakers were to vary their pitch in a non-standard way, it could affect the production of happiness (which requires pitch modulation) more than the production of anger (which could be expressed with either a wide or small pitch range). This hypothesis should be directly tested in future studies.

A final point regarding the specific effect observed for fear expressions is worth noting. In the literature on facial expression recognition, it has been repeatedly suggested that individuals with HFA process fearful expressions differently from NT individuals [36, 37], with associated differences in brain activation patterns. Based on the findings of our study, it can be speculated that these processing differences extend to the speech channel as well, suggesting modality (e.g. vocal, facial, gesture) non-specific effects.

### Compensation effect?

To the best of our knowledge, this is the first study to consider gender effects when examining whether the emotional expressions of ASD and NT speakers are recognized differently by NT listeners. The finding that emotional expressions from female ASD speakers are well recognized suggests that productions generally align with the expectations of listeners. This could indicate that female autistic speakers do not exhibit atypical emotional prosody or that they make an effort to imitate NT behavior. There is a growing body of evidence suggesting that autistic individuals, particularly females, engage in compensatory behaviors and attempt to conceal their ASD symptoms in order to fit in with NT individuals [17, 38–40]. This camouflaging behavior is thought to contribute to the later diagnosis or even misdiagnosis of autistic females [41–45]. While the current data do not provide specific evidence of imitating NT expressions by female speakers, the acoustic analyses of our sample indicate that there are differences in speech expression (less variation in loudness and reduced vocal effort), regardless of the emotion. This is consistent with developmental studies, which have shown atypical prosodic expressions in ASD (e.g., [32]), although girls were often underrepresented in those samples. Here, ASD speakers were perceived as sounding more monotone than NT speakers, suggesting that the reduced loudness variation in autistic female speakers is detectable by NT listeners, even if it does not lead to difficulties in recognizing emotional intentions.

Yet, the current findings highlight that female individuals with ASD largely adhere to (NT) display rules for emotional behavior in the vocal domain (e.g., [8]), except when expressing fear. This could mean that female autistic speakers develop compensatory vocal strategies during social communication to a greater extent than their male counterparts, a finding observed in other emotional domains (e.g., forcing eye contact or displaying expected emotional facial expressions; [17]). This effect is noteworthy as speakers were asked to express emotions in a manner *they* deemed appropriate, though the instruction itself may not be enough to overcome a desire to express stimuli in line with what they believed the experimenter expected. This should be directly investigated by providing explicit instructions that compensation strategies are not anticipated. Similarly, future research could directly examine the influence of camouflaging strategies on prosody production by administering the Camouflaging Autistic Traits Questionnaire (CAT-Q; [46]) and correlating the results with prosodic patterns.

### Severity of ASD

An alternative explanation for the observed sex effects may be that differnces in production stem from differences in the severity of ASD. Although the AQ scores obtained in this study were similar, and there is currently no research employing AQ to predict ASD severity, it would be valuable for future studies to explore alternative approaches to assess HFA populations and ascertain the role of ASD severity in these differences. Future studies should thus aim to even further increase number of female and male speakers. Here, we aimed to present several exemplars from speakers but future studies could increase speaker size by reducing exemplars needed from each speaker.

### Clinical relevance

The current findings have relevance for autism assessment and diagnosis. It is essential to consider that female ASD speakers exhibit distinct patterns of prosody production compared to their male counterparts. Whether these patterns result from mimicking prosody from NT speakers as a compensatory strategy or simply displaying less divergent patterns, the resemblance of prosody production to that of NT speakers can potentially hinder accurate and timely diagnosis, particularly for individuals with HFA (see [47]). It is hoped that the present results will prompt future studies to systematically investigate these behaviors in larger sample sizes, while also controlling for other characteristics. For instance, although age was matched between ASD and NT speakers in this study, it is important for future research to carefully consider and control for potential age effects (though note prior evidence indicates successful recognition of emotional prosody from both young and middle-aged speakers (e.g., [24]).

### Conclusion

This study is the first to document differences in the perception of vocal emotions by NT listeners when expressed by both male and female ASD speakers. It emphasizes the importance of studying emotional prosody not only in male ASD speakers but also in females. Future investigations should aim to move beyond simple comparisons of emotion recognition and instead delve into understanding the underlying mechanisms through which non-prototypical cue use and a lack of control over voice cues may contribute to difficulties in social interactions. Additionally, the potential impact of camouflaging strategies on emotional speech output should be further explored.

## Supporting information

S1 Table Results of acoustic analysis of materials used. (DOCX)

S2 Table Pairwise comparisons main effect Study 1. (DOCX)

S3 Table Pairwise comparisons 3-way interaction Study 1. (DOCX)

S4 Table Summary of significant and non-significant main effects and interactions for valence ratings Study 2. (DOCX)

S5 Table Summary of significant and non-significant main effects and interactions for voice modulation ratings Study 2. (DOCX)

S6 Table Summary of significant and non-significant main effects and interactions for voice control ratings Study 2. (DOCX)

S7 Table Pairwise comparisons valence rating, emotion main effect Study 2. (DOCX)

S8 Table Valence rating: Pairwise comparison 3-way interaction Study 2. (DOCX)

S9 Table . Pairwise comparisons voice modulation rating, emotion main effect Study 2. (DOCX)

S10 Table Voice modulation rating: Pairwise comparison Study 2. (DOCX)

S11 Table Voice control rating: Pairwise comparison, main effect emotion Study 2. (DOCX)

S12 Table Voice control rating: Pairwise comparison Study 2. (DOCX)
