# Supplementary material for: How neurotypical listeners recognize emotions expressed through vocal cues by speakers with high-functioning autism
Source: PLoS One. 2023 Oct 24;18(10):e0293233. doi: 10.1371/journal.pone.0293233 (PMC10597502; doi:10.1371/journal.pone.0293233)
Supplement: S1 Table — (DOCX) [file pone.0293233.s001.docx]

***Supplementary Materials:***

**S1 Table. Results of Acoustic Analysis of Materials Used**

| Speaker Type | Mean pitch (ST) | Standard deviation pitch (ST) | Range pitch (ST) | Duration (seconds) | Energy in high frequency band (dB) | Standard deviation amplitude (dB) | Range amplitude (dB) |
| --- | --- | --- | --- | --- | --- | --- | --- |
| ASD Female | 13.7 | 3.6 | 13.6 | 2.6 | 17.3 | 6.5 | 25.8 |
| NT Female | 14.1 | 2.8 | 12.2 | 2.4 | 25.4 | 8.9 | 34.4 |
| ASD Male | 5.4 | 3.2 | 13.0 | 3.0 | 19.5 | 7.6 | 28.4 |
| NT Male | 2.3 | 2.7 | 11.5 | 2.3 | 22.8 | 7.5 | 30.3 |
