# Supplementary material for: How neurotypical listeners recognize emotions expressed through vocal cues by speakers with high-functioning autism
Source: PLoS One. 2023 Oct 24;18(10):e0293233. doi: 10.1371/journal.pone.0293233 (PMC10597502; doi:10.1371/journal.pone.0293233)
Supplement: S2 Table — (DOCX) [file pone.0293233.s002.docx]

**S2 Table. Pairwise comparisons main effect Study 1**

| **Pairwise Comparisons: Emotion** | | | | | | |
| --- | --- | --- | --- | --- | --- | --- |
| **Measure: Hu Scores** | | | | | | |
| (I) Emotion | (J) Emotion | Mean Difference (I-J) | Std. Error | Sig.^b^ | 95% Confidence Interval for Difference^b^ | |
|  |  |  |  |  | Lower Bound | Upper Bound |
| Anger | Fear | .181^*^ | .027 | .000 | .128 | .235 |
|  | Happiness | .252^*^ | .025 | .000 | .202 | .301 |
|  | Neutral | .178^*^ | .022 | .000 | .133 | .224 |
|  | Sadness | .199^*^ | .026 | .000 | .146 | .251 |
|  | Surprise | .132^*^ | .024 | .000 | .084 | .180 |
| Fear | Anger | -.181^*^ | .027 | .000 | -.235 | -.128 |
|  | Happiness | .070^*^ | .024 | .004 | .023 | .118 |
|  | Neutral | -.003 | .028 | .921 | -.058 | .053 |
|  | Sadness | .017 | .021 | .410 | -.025 | .060 |
|  | Surprise | -.049^*^ | .024 | .048 | -.098 | .000 |
| Happiness | Anger | -.252^*^ | .025 | .000 | -.301 | -.202 |
|  | Fear | -.070^*^ | .024 | .004 | -.118 | -.023 |
|  | Neutral | -.073^*^ | .020 | .001 | -.113 | -.034 |
|  | Sadness | -.053^*^ | .021 | .016 | -.096 | -.010 |
|  | Surprise | -.120^*^ | .017 | .000 | -.155 | -.085 |
| Neutral | Anger | -.178^*^ | .022 | .000 | -.224 | -.133 |
|  | Fear | .003 | .028 | .921 | -.053 | .058 |
|  | Happiness | .073^*^ | .020 | .001 | .034 | .113 |
|  | Sadness | .020 | .025 | .417 | -.029 | .070 |
|  | Surprise | -.047^*^ | .020 | .023 | -.086 | -.007 |
| Sadness | Anger | -.199^*^ | .026 | .000 | -.251 | -.146 |
|  | Fear | -.017 | .021 | .410 | -.060 | .025 |
|  | Happiness | .053^*^ | .021 | .016 | .010 | .096 |
|  | Neutral | -.020 | .025 | .417 | -.070 | .029 |
|  | Surprise | -.067^*^ | .022 | .003 | -.110 | -.023 |
| Surprise | Anger | -.132^*^ | .024 | .000 | -.180 | -.084 |
|  | Fear | .049^*^ | .024 | .048 | .000 | .098 |
|  | Happiness | .120^*^ | .017 | .000 | .085 | .155 |
|  | Neutral | .047^*^ | .020 | .023 | .007 | .086 |
|  | Sadness | .067^*^ | .022 | .003 | .023 | .110 |
| Based on estimated marginal means | | | | | | |
| *. The mean difference is significant at the .05 level. | | | | | | |
| b. Adjustment for multiple comparisons: Least Significant Difference (equivalent to no adjustments). | | | | | | |
