# Supplementary material for: How neurotypical listeners recognize emotions expressed through vocal cues by speakers with high-functioning autism
Source: PLoS One. 2023 Oct 24;18(10):e0293233. doi: 10.1371/journal.pone.0293233 (PMC10597502; doi:10.1371/journal.pone.0293233)
Supplement: S3 Table — (DOCX) [file pone.0293233.s003.docx]

**S3 Table. Pairwise comparisons 3-way interaction Study 1**

| **Pairwise Comparisons: Speaker Sex * Emotion * Speaker Type** | | | | | | | | |
| --- | --- | --- | --- | --- | --- | --- | --- | --- |
| **Measure: Hu Scores** | | | | | | | | |
| Speaker Sex | Emotion | (I) Speaker Type | (J) Speaker Type | Mean Difference (I-J) | Std. Error | Sig.^b^ | 95% Confidence Interval for Difference^b^ | |
|  |  |  |  |  |  |  | Lower Bound | Upper Bound |
| Female | Anger | ASD | NT | -.143 | .096 | .144 | -.336 | .050 |
|  |  | NT | ASD | .143 | .096 | .144 | -.050 | .336 |
|  | Fear | ASD | NT | -.296^*^ | .068 | .000 | -.433 | -.158 |
|  |  | NT | ASD | .296^*^ | .068 | .000 | .158 | .433 |
|  | Happiness | ASD | NT | -.074 | .048 | .128 | -.171 | .022 |
|  |  | NT | ASD | .074 | .048 | .128 | -.022 | .171 |
|  | Neutral | ASD | NT | -.071 | .051 | .168 | -.173 | .031 |
|  |  | NT | ASD | .071 | .051 | .168 | -.031 | .173 |
|  | Sadness | ASD | NT | .001 | .051 | .984 | -.101 | .103 |
|  |  | NT | ASD | -.001 | .051 | .984 | -.103 | .101 |
|  | Surprise | ASD | NT | -.127 | .066 | .062 | -.260 | .007 |
|  |  | NT | ASD | .127 | .066 | .062 | -.007 | .260 |
| Male | Anger | ASD | NT | -.129^*^ | .032 | .000 | -.193 | -.066 |
|  |  | NT | ASD | .129^*^ | .032 | .000 | .066 | .193 |
|  | Fear | ASD | NT | -.318^*^ | .038 | .000 | -.393 | -.242 |
|  |  | NT | ASD | .318^*^ | .038 | .000 | .242 | .393 |
|  | Happiness | ASD | NT | -.263^*^ | .029 | .000 | -.320 | -.205 |
|  |  | NT | ASD | .263^*^ | .029 | .000 | .205 | .320 |
|  | Neutral | ASD | NT | .123^*^ | .029 | .000 | .065 | .181 |
|  |  | NT | ASD | -.123^*^ | .029 | .000 | -.181 | -.065 |
|  | Sadness | ASD | NT | -.079^*^ | .035 | .026 | -.149 | -.010 |
|  |  | NT | ASD | .079^*^ | .035 | .026 | .010 | .149 |
|  | Surprise | ASD | NT | -.192^*^ | .043 | .000 | -.278 | -.107 |
|  |  | NT | ASD | .192^*^ | .043 | .000 | .107 | .278 |
| Based on estimated marginal means | | | | | | | | |
| *. The mean difference is significant at the .05 level. | | | | | | | | |
| b. Adjustment for multiple comparisons: Least Significant Difference (equivalent to no adjustments). | | | | | | | | |
