# Supplementary material for: How neurotypical listeners recognize emotions expressed through vocal cues by speakers with high-functioning autism
Source: PLoS One. 2023 Oct 24;18(10):e0293233. doi: 10.1371/journal.pone.0293233 (PMC10597502; doi:10.1371/journal.pone.0293233)
Supplement: S4 Table — (DOCX) [file pone.0293233.s004.docx]

**S4 Table. Summary of significant and non-significant main effects and interactions for valence ratings Study 2**

| **Factors** | **df** | **F -Value** | **Significance** | **Effect size (partial Eta squared)** |
| --- | --- | --- | --- | --- |
| Speaker Sex | 1,23 | 4.857 | .038 | .174 |
| Speaker Type | 1,23 | 1.260 | .273 | .052 |
| Emotion | 5,115 | 42.678 | <.00005 | .650 |
| Speaker Sex * Speaker Type | 1,23 | .170 | .684 | .007 |
| Speaker Sex * Emotion | 5,115 | 13.783 | <.00005 | .375 |
| Speaker Type * Emotion | 5,115 | 9.931 | <.00005 | .302 |
| Speaker Type * Speaker Type* Emotion | 5,115 | 2.660 | .026 | .104 |
