# Supplementary material for: How neurotypical listeners recognize emotions expressed through vocal cues by speakers with high-functioning autism
Source: PLoS One. 2023 Oct 24;18(10):e0293233. doi: 10.1371/journal.pone.0293233 (PMC10597502; doi:10.1371/journal.pone.0293233)
Supplement: S5 Table — (DOCX) [file pone.0293233.s005.docx]

**S5 Table. Summary of significant and non-significant main effects and interactions for voice modulation ratings Study 2**

| **Factors** | **df** | **F -Value** | **Significance** | **Effect size (partial Eta squared)** |
| --- | --- | --- | --- | --- |
| Speaker Sex | 1,23 | 33.205 | <.00005 | .591 |
| Speaker Type | 1,23 | .004 | .949 | .0 |
| Emotion | 5,115 | 42.468 | <.00005 | .591 |
| Speaker Sex * Speaker Type | 1,23 | .549 | .466 | .023 |
| Speaker Sex * Emotion | 5,115 | 3.565 | .005 | .134 |
| Speaker Type * Emotion | 5,115 | 7.824 | <.00005 | .254 |
| Speaker Type * Speaker Type* Emotion | 5,115 | 1.819 | .114 | .073 |
