# Supplementary material for: How neurotypical listeners recognize emotions expressed through vocal cues by speakers with high-functioning autism
Source: PLoS One. 2023 Oct 24;18(10):e0293233. doi: 10.1371/journal.pone.0293233 (PMC10597502; doi:10.1371/journal.pone.0293233)
Supplement: S6 Table — (DOCX) [file pone.0293233.s006.docx]

**S6 Table. Summary of significant and non-significant main effects and interactions for voice control ratings Study 2**

| **Factors** | **df** | **F -Value** | **Significance** | **Effect size (partial Eta squared)** |
| --- | --- | --- | --- | --- |
| Speaker Sex | 1,23 | .893 | .354 | .037 |
| Speaker Type | 1,23 | 29.785 | <.00005 | .564 |
| Emotion | 5,115 | 12.647 | <.00005 | .355 |
| Speaker Sex * Speaker Type | 1,23 | 29.188 |  | .559 |
| Speaker Sex * Emotion | 5,115 | 6.664 | <.00005 | .225 |
| Speaker Type * Emotion | 5,115 | 6.899 | <.00005 | .231 |
| Speaker Type * Speaker Type* Emotion | 5,115 | 3.820 | .003 | .142 |
