# Supplementary material for: How neurotypical listeners recognize emotions expressed through vocal cues by speakers with high-functioning autism
Source: PLoS One. 2023 Oct 24;18(10):e0293233. doi: 10.1371/journal.pone.0293233 (PMC10597502; doi:10.1371/journal.pone.0293233)
Supplement: S7 Table — (DOCX) [file pone.0293233.s007.docx]

**S7 Table. Pairwise comparisons Valence Rating, Emotion main effect Study 2**

| **Pairwise Comparisons: Emotion** | | | | | | |
| --- | --- | --- | --- | --- | --- | --- |
| Measure: Valence Rating | | | | | | |
| (I) Emotion | (J) Emotion | Mean Difference (I-J) | Std. Error | Sig.^b^ | 95% Confidence Interval for Difference^b^ | |
|  |  |  |  |  | Lower Bound | Upper Bound |
| Anger | Fear | -1.253^*^ | .167 | .000 | -1.599 | -.907 |
|  | Happiness | -.240 | .116 | .051 | -.480 | .001 |
|  | Neutral | -.101 | .106 | .355 | -.321 | .120 |
|  | Sadness | -.131 | .125 | .308 | -.390 | .128 |
|  | Surprise | -1.381^*^ | .152 | .000 | -1.696 | -1.066 |
| Fear | Anger | 1.253^*^ | .167 | .000 | .907 | 1.599 |
|  | Happiness | 1.013^*^ | .153 | .000 | .697 | 1.329 |
|  | Neutral | 1.152^*^ | .177 | .000 | .786 | 1.518 |
|  | Sadness | 1.122^*^ | .139 | .000 | .834 | 1.410 |
|  | Surprise | -.128 | .078 | .113 | -.289 | .033 |
| Happiness | Anger | .240 | .116 | .051 | -.001 | .480 |
|  | Fear | -1.013^*^ | .153 | .000 | -1.329 | -.697 |
|  | Neutral | .139 | .078 | .086 | -.021 | .300 |
|  | Sadness | .109 | .105 | .309 | -.108 | .326 |
|  | Surprise | -1.141^*^ | .155 | .000 | -1.461 | -.821 |
| Neutral | Anger | .101 | .106 | .355 | -.120 | .321 |
|  | Fear | -1.152^*^ | .177 | .000 | -1.518 | -.786 |
|  | Happiness | -.139 | .078 | .086 | -.300 | .021 |
|  | Sadness | -.030 | .141 | .833 | -.322 | .262 |
|  | Surprise | -1.280^*^ | .160 | .000 | -1.611 | -.950 |
| Sadness | Anger | .131 | .125 | .308 | -.128 | .390 |
|  | Fear | -1.122^*^ | .139 | .000 | -1.410 | -.834 |
|  | Happiness | -.109 | .105 | .309 | -.326 | .108 |
|  | Neutral | .030 | .141 | .833 | -.262 | .322 |
|  | Surprise | -1.250^*^ | .129 | .000 | -1.516 | -.984 |
| Surprise | Anger | 1.381^*^ | .152 | .000 | 1.066 | 1.696 |
|  | Fear | .128 | .078 | .113 | -.033 | .289 |
|  | Happiness | 1.141^*^ | .155 | .000 | .821 | 1.461 |
|  | Neutral | 1.280^*^ | .160 | .000 | .950 | 1.611 |
|  | Sadness | 1.250^*^ | .129 | .000 | .984 | 1.516 |
| Based on estimated marginal means | | | | | | |
| *. The mean difference is significant at the .05 level. | | | | | | |
| b. Adjustment for multiple comparisons: Least Significant Difference (equivalent to no adjustments). | | | | | | |
