# Supplementary material for: How neurotypical listeners recognize emotions expressed through vocal cues by speakers with high-functioning autism
Source: PLoS One. 2023 Oct 24;18(10):e0293233. doi: 10.1371/journal.pone.0293233 (PMC10597502; doi:10.1371/journal.pone.0293233)
Supplement: S8 Table — (DOCX) [file pone.0293233.s008.docx]

**S8 Table. Valence Rating: pairwise comparison 3-way interaction Study 2**

| **Pairwise Comparisons: Speaker Sex * Emotion * Speaker Type** | | | | | | | | |
| --- | --- | --- | --- | --- | --- | --- | --- | --- |
| **Measure: Valence Rating** | | | | | | | | |
| Speaker Sex | Emotion | (I) Speaker Type | (J) Speaker Type | Mean Difference (I-J) | Std. Error | Sig.^b^ | 95% Confidence Interval for Difference^b^ | |
|  |  |  |  |  |  |  | Lower Bound | Upper Bound |
| Female | Anger | ASD | NT | .113 | .200 | .576 | -.300 | .526 |
|  |  | NT | ASD | -.113 | .200 | .576 | -.526 | .300 |
|  | Fear | ASD | NT | -.002 | .177 | .990 | -.369 | .364 |
|  |  | NT | ASD | .002 | .177 | .990 | -.364 | .369 |
|  | Happiness | ASD | NT | .218 | .162 | .193 | -.118 | .554 |
|  |  | NT | ASD | -.218 | .162 | .193 | -.554 | .118 |
|  | Neutral | ASD | NT | -.571^*^ | .151 | .001 | -.883 | -.259 |
|  |  | NT | ASD | .571^*^ | .151 | .001 | .259 | .883 |
|  | Sadness | ASD | NT | .520^*^ | .222 | .028 | .060 | .979 |
|  |  | NT | ASD | -.520^*^ | .222 | .028 | -.979 | -.060 |
|  | Surprise | ASD | NT | -.774^*^ | .217 | .002 | -1.223 | -.325 |
|  |  | NT | ASD | .774^*^ | .217 | .002 | .325 | 1.223 |
| Male | Anger | ASD | NT | .136 | .152 | .380 | -.178 | .450 |
|  |  | NT | ASD | -.136 | .152 | .380 | -.450 | .178 |
|  | Fear | ASD | NT | -.478^*^ | .191 | .020 | -.872 | -.083 |
|  |  | NT | ASD | .478^*^ | .191 | .020 | .083 | .872 |
|  | Happiness | ASD | NT | .214 | .152 | .172 | -.100 | .528 |
|  |  | NT | ASD | -.214 | .152 | .172 | -.528 | .100 |
|  | Neutral | ASD | NT | .131 | .146 | .377 | -.170 | .433 |
|  |  | NT | ASD | -.131 | .146 | .377 | -.433 | .170 |
|  | Sadness | ASD | NT | .142 | .179 | .435 | -.228 | .512 |
|  |  | NT | ASD | -.142 | .179 | .435 | -.512 | .228 |
|  | Surprise | ASD | NT | -.360^*^ | .168 | .042 | -.707 | -.014 |
|  |  | NT | ASD | .360^*^ | .168 | .042 | .014 | .707 |
| Based on estimated marginal means | | | | | | | | |
| *. The mean difference is significant at the .05 level. | | | | | | | | |
| b. Adjustment for multiple comparisons: Least Significant Difference (equivalent to no adjustments). | | | | | | | | |
