# Supplementary material for: How neurotypical listeners recognize emotions expressed through vocal cues by speakers with high-functioning autism
Source: PLoS One. 2023 Oct 24;18(10):e0293233. doi: 10.1371/journal.pone.0293233 (PMC10597502; doi:10.1371/journal.pone.0293233)
Supplement: S9 Table — (DOCX) [file pone.0293233.s009.docx]

**S9 Table. Pairwise comparisons Voice Modulation Rating, Emotion main effect Study 2**

| **Pairwise Comparisons: Emotion** | | | | | | |
| --- | --- | --- | --- | --- | --- | --- |
| Measure: Modulation Rating | | | | | | |
| (I) Emotion | (J) Emotion | Mean Difference (I-J) | Std. Error | Sig.^b^ | 95% Confidence Interval for Difference^b^ | |
|  |  |  |  |  | Lower Bound | Upper Bound |
| Anger | Fear | -.557^*^ | .093 | .000 | -.749 | -.364 |
|  | Happiness | .678^*^ | .197 | .002 | .270 | 1.086 |
|  | Neutral | .807^*^ | .181 | .000 | .433 | 1.181 |
|  | Sadness | -.025 | .088 | .779 | -.208 | .158 |
|  | Surprise | -.889^*^ | .106 | .000 | -1.108 | -.670 |
| Fear | Anger | .557^*^ | .093 | .000 | .364 | .749 |
|  | Happiness | 1.235^*^ | .228 | .000 | .763 | 1.706 |
|  | Neutral | 1.364^*^ | .219 | .000 | .911 | 1.818 |
|  | Sadness | .532^*^ | .106 | .000 | .313 | .750 |
|  | Surprise | -.332^*^ | .102 | .004 | -.543 | -.121 |
| Happiness | Anger | -.678^*^ | .197 | .002 | -1.086 | -.270 |
|  | Fear | -1.235^*^ | .228 | .000 | -1.706 | -.763 |
|  | Neutral | .129 | .075 | .099 | -.026 | .285 |
|  | Sadness | -.703^*^ | .197 | .002 | -1.110 | -.296 |
|  | Surprise | -1.567^*^ | .227 | .000 | -2.037 | -1.096 |
| Neutral | Anger | -.807^*^ | .181 | .000 | -1.181 | -.433 |
|  | Fear | -1.364^*^ | .219 | .000 | -1.818 | -.911 |
|  | Happiness | -.129 | .075 | .099 | -.285 | .026 |
|  | Sadness | -.832^*^ | .180 | .000 | -1.206 | -.459 |
|  | Surprise | -1.696^*^ | .205 | .000 | -2.121 | -1.271 |
| Sadness | Anger | .025 | .088 | .779 | -.158 | .208 |
|  | Fear | -.532^*^ | .106 | .000 | -.750 | -.313 |
|  | Happiness | .703^*^ | .197 | .002 | .296 | 1.110 |
|  | Neutral | .832^*^ | .180 | .000 | .459 | 1.206 |
|  | Surprise | -.864^*^ | .092 | .000 | -1.054 | -.674 |
| Surprise | Anger | .889^*^ | .106 | .000 | .670 | 1.108 |
|  | Fear | .332^*^ | .102 | .004 | .121 | .543 |
|  | Happiness | 1.567^*^ | .227 | .000 | 1.096 | 2.037 |
|  | Neutral | 1.696^*^ | .205 | .000 | 1.271 | 2.121 |
|  | Sadness | .864^*^ | .092 | .000 | .674 | 1.054 |
| Based on estimated marginal means | | | | | | |
| *. The mean difference is significant at the .05 level. | | | | | | |
| b. Adjustment for multiple comparisons: Least Significant Difference (equivalent to no adjustments). | | | | | | |
