# Supplementary material for: How neurotypical listeners recognize emotions expressed through vocal cues by speakers with high-functioning autism
Source: PLoS One. 2023 Oct 24;18(10):e0293233. doi: 10.1371/journal.pone.0293233 (PMC10597502; doi:10.1371/journal.pone.0293233)
Supplement: S10 Table — (DOCX) [file pone.0293233.s010.docx]

**S10 Table. Voice Modulation Rating: pairwise comparison Study 2**

| **Pairwise Comparisons: Speaker Sex * Emotion * Speaker Type** | | | | | | | | |
| --- | --- | --- | --- | --- | --- | --- | --- | --- |
| **Measure: Modulation Rating** | | | | | | | | |
| Speaker Sex | Emotion | **(I) Speaker Type** | (J) Speaker Type | Mean Difference (I-J) | Std. Error | Sig.^b^ | 95% Confidence Interval for Difference^b^ | |
|  |  |  |  |  |  |  | Lower Bound | Upper Bound |
| Female | Anger | ASD | NT | .205 | .178 | .261 | -.163 | .573 |
|  |  | NT | ASD | -.205 | .178 | .261 | -.573 | .163 |
|  | Fear | ASD | NT | -.027 | .130 | .836 | -.296 | .242 |
|  |  | NT | ASD | .027 | .130 | .836 | -.242 | .296 |
|  | Happiness | ASD | NT | .197 | .230 | .400 | -.279 | .673 |
|  |  | NT | ASD | -.197 | .230 | .400 | -.673 | .279 |
|  | Neutral | ASD | NT | -.331 | .191 | .097 | -.726 | .065 |
|  |  | NT | ASD | .331 | .191 | .097 | -.065 | .726 |
|  | Sadness | ASD | NT | .498^*^ | .161 | .005 | .164 | .832 |
|  |  | NT | ASD | -.498^*^ | .161 | .005 | -.832 | -.164 |
|  | Surprise | ASD | NT | -.335 | .165 | .053 | -.676 | .005 |
|  |  | NT | ASD | .335 | .165 | .053 | -.005 | .676 |
| Male | Anger | ASD | NT | .052 | .144 | .722 | -.245 | .349 |
|  |  | NT | ASD | -.052 | .144 | .722 | -.349 | .245 |
|  | Fear | ASD | NT | -.543^*^ | .173 | .005 | -.902 | -.185 |
|  |  | NT | ASD | .543^*^ | .173 | .005 | .185 | .902 |
|  | Happiness | ASD | NT | .429^*^ | .178 | .025 | .060 | .798 |
|  |  | NT | ASD | -.429^*^ | .178 | .025 | -.798 | -.060 |
|  | Neutral | ASD | NT | .057 | .153 | .714 | -.260 | .373 |
|  |  | NT | ASD | -.057 | .153 | .714 | -.373 | .260 |
|  | Sadness | ASD | NT | .233 | .120 | .065 | -.015 | .482 |
|  |  | NT | ASD | -.233 | .120 | .065 | -.482 | .015 |
|  | Surprise | ASD | NT | -.470^*^ | .161 | .008 | -.803 | -.138 |
|  |  | NT | ASD | .470^*^ | .161 | .008 | .138 | .803 |
| Based on estimated marginal means | | | | | | | | |
| *. The mean difference is significant at the .05 level. | | | | | | | | |
| b. Adjustment for multiple comparisons: Least Significant Difference (equivalent to no adjustments). | | | | | | | | |
