# Supplementary material for: How neurotypical listeners recognize emotions expressed through vocal cues by speakers with high-functioning autism
Source: PLoS One. 2023 Oct 24;18(10):e0293233. doi: 10.1371/journal.pone.0293233 (PMC10597502; doi:10.1371/journal.pone.0293233)
Supplement: S11 Table — (DOCX) [file pone.0293233.s011.docx]

**S11 Table. Voice Control Rating: pairwise comparison, main effect Emotion Study 2**

| **Pairwise Comparisons: Emotion** | | | | | | |
| --- | --- | --- | --- | --- | --- | --- |
| Measure: Voice Control | | | | | | |
| (I) Emotion | (J) Emotion | Mean Difference (I-J) | Std. Error | Sig.^b^ | 95% Confidence Interval for Difference^b^ | |
|  |  |  |  |  | Lower Bound | Upper Bound |
| Anger | Fear | -.477^*^ | .117 | .000 | -.719 | -.234 |
|  | Happiness | -.442^*^ | .114 | .001 | -.677 | -.206 |
|  | Neutral | .040 | .101 | .695 | -.170 | .250 |
|  | Sadness | .241 | .124 | .065 | -.016 | .498 |
|  | Surprise | -.147 | .097 | .144 | -.349 | .054 |
| Fear | Anger | .477^*^ | .117 | .000 | .234 | .719 |
|  | Happiness | .035 | .111 | .755 | -.194 | .264 |
|  | Neutral | .517^*^ | .127 | .000 | .253 | .780 |
|  | Sadness | .717^*^ | .143 | .000 | .423 | 1.012 |
|  | Surprise | .329^*^ | .087 | .001 | .149 | .510 |
| Happiness | Anger | .442^*^ | .114 | .001 | .206 | .677 |
|  | Fear | -.035 | .111 | .755 | -.264 | .194 |
|  | Neutral | .482^*^ | .091 | .000 | .293 | .671 |
|  | Sadness | .682^*^ | .103 | .000 | .469 | .895 |
|  | Surprise | .294^*^ | .122 | .024 | .042 | .547 |
| Neutral | Anger | -.040 | .101 | .695 | -.250 | .170 |
|  | Fear | -.517^*^ | .127 | .000 | -.780 | -.253 |
|  | Happiness | -.482^*^ | .091 | .000 | -.671 | -.293 |
|  | Sadness | .200 | .098 | .053 | -.003 | .404 |
|  | Surprise | -.188 | .108 | .096 | -.411 | .036 |
| Sadness | Anger | -.241 | .124 | .065 | -.498 | .016 |
|  | Fear | -.717^*^ | .143 | .000 | -1.012 | -.423 |
|  | Happiness | -.682^*^ | .103 | .000 | -.895 | -.469 |
|  | Neutral | -.200 | .098 | .053 | -.404 | .003 |
|  | Surprise | -.388^*^ | .129 | .006 | -.654 | -.121 |
| Surprise | Anger | .147 | .097 | .144 | -.054 | .349 |
|  | Fear | -.329^*^ | .087 | .001 | -.510 | -.149 |
|  | Happiness | -.294^*^ | .122 | .024 | -.547 | -.042 |
|  | Neutral | .188 | .108 | .096 | -.036 | .411 |
|  | Sadness | .388^*^ | .129 | .006 | .121 | .654 |
| Based on estimated marginal means | | | | | | |
| *. The mean difference is significant at the .05 level. | | | | | | |
| b. Adjustment for multiple comparisons: Least Significant Difference (equivalent to no adjustments). | | | | | | |
