# Supplementary material for: How neurotypical listeners recognize emotions expressed through vocal cues by speakers with high-functioning autism
Source: PLoS One. 2023 Oct 24;18(10):e0293233. doi: 10.1371/journal.pone.0293233 (PMC10597502; doi:10.1371/journal.pone.0293233)
Supplement: S12 Table — (DOCX) [file pone.0293233.s012.docx]

**S12 Table. Voice Control Rating: pairwise comparison Study 2**

| **Pairwise Comparisons: Speaker Sex * Emotion * Speaker Type** | | | | | | | | |
| --- | --- | --- | --- | --- | --- | --- | --- | --- |
| **Measure: Voice Control Rating** | | | | | | | | |
| SpeakerSex | Emotion | (I) SpeakerType | (J) SpeakerType | Mean Difference (I-J) | Std. Error | Sig.^b^ | 95% Confidence Interval for Difference^b^ | |
|  |  |  |  |  |  |  | Lower Bound | Upper Bound |
| Female | Anger | ASD | NT | -.337 | .216 | .133 | -.785 | .111 |
|  |  | NT | ASD | .337 | .216 | .133 | -.111 | .785 |
|  | Fear | ASD | NT | -.112 | .114 | .335 | -.347 | .123 |
|  |  | NT | ASD | .112 | .114 | .335 | -.123 | .347 |
|  | Happiness | ASD | NT | .459^*^ | .183 | .020 | .080 | .838 |
|  |  | NT | ASD | -.459^*^ | .183 | .020 | -.838 | -.080 |
|  | Neutral | ASD | NT | -.429^*^ | .178 | .024 | -.796 | -.061 |
|  |  | NT | ASD | .429^*^ | .178 | .024 | .061 | .796 |
|  | Sadness | ASD | NT | .288 | .232 | .227 | -.192 | .767 |
|  |  | NT | ASD | -.288 | .232 | .227 | -.767 | .192 |
|  | Surprise | ASD | NT | .754^*^ | .245 | .005 | .248 | 1.260 |
|  |  | NT | ASD | -.754^*^ | .245 | .005 | -1.260 | -.248 |
| Male | Anger | ASD | NT | -.817^*^ | .145 | .000 | -1.117 | -.517 |
|  |  | NT | ASD | .817^*^ | .145 | .000 | .517 | 1.117 |
|  | Fear | ASD | NT | -.847^*^ | .173 | .000 | -1.205 | -.490 |
|  |  | NT | ASD | .847^*^ | .173 | .000 | .490 | 1.205 |
|  | Happiness | ASD | NT | -.164 | .144 | .267 | -.461 | .134 |
|  |  | NT | ASD | .164 | .144 | .267 | -.134 | .461 |
|  | Neutral | ASD | NT | -1.035^*^ | .200 | .000 | -1.448 | -.622 |
|  |  | NT | ASD | 1.035^*^ | .200 | .000 | .622 | 1.448 |
|  | Sadness | ASD | NT | -.741^*^ | .178 | .000 | -1.110 | -.373 |
|  |  | NT | ASD | .741^*^ | .178 | .000 | .373 | 1.110 |
|  | Surprise | ASD | NT | -1.037^*^ | .217 | .000 | -1.485 | -.589 |
|  |  | NT | ASD | 1.037^*^ | .217 | .000 | .589 | 1.485 |
| Based on estimated marginal means | | | | | | | | |
| *. The mean difference is significant at the .05 level. | | | | | | | | |
| b. Adjustment for multiple comparisons: Least Significant Difference (equivalent to no adjustments). | | | | | | | | |
